# Supplementary figures and images for: Identifying liver metastasis-related hub genes in breast cancer and characterizing SPARCL1 as a potential prognostic biomarker
Source: PeerJ. 2023 May 8;11:e15311. doi: 10.7717/peerj.15311 (PMC10174054; doi:10.7717/peerj.15311)

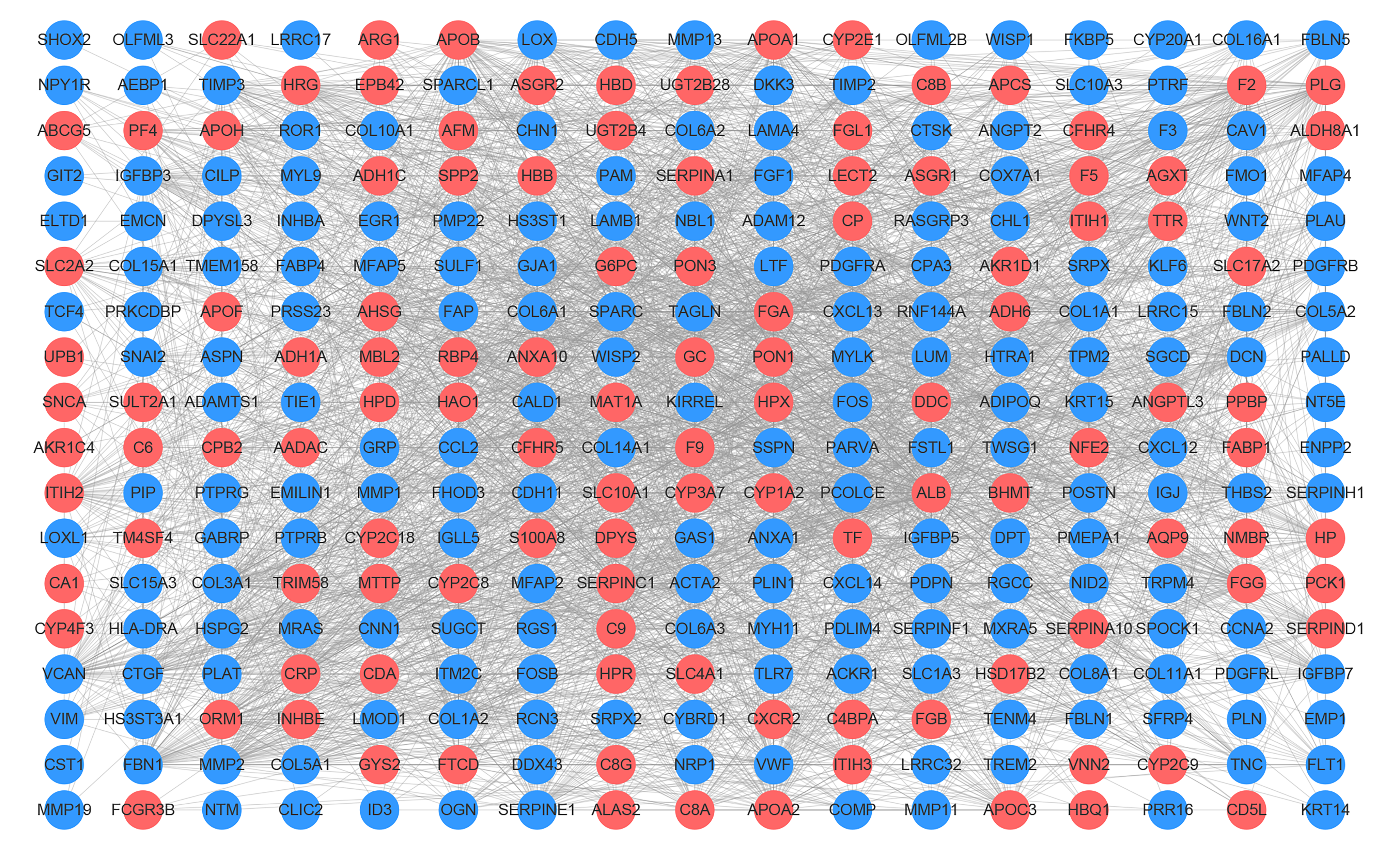

Supplement: Supplemental Information 2 — Each node represents one gene, and the edges between nodes represent interactions. Blue and red indicate down- and up-regulated DEGs, respectively. PPI: protein-protein interaction [file peerj-11-15311-s002.png]

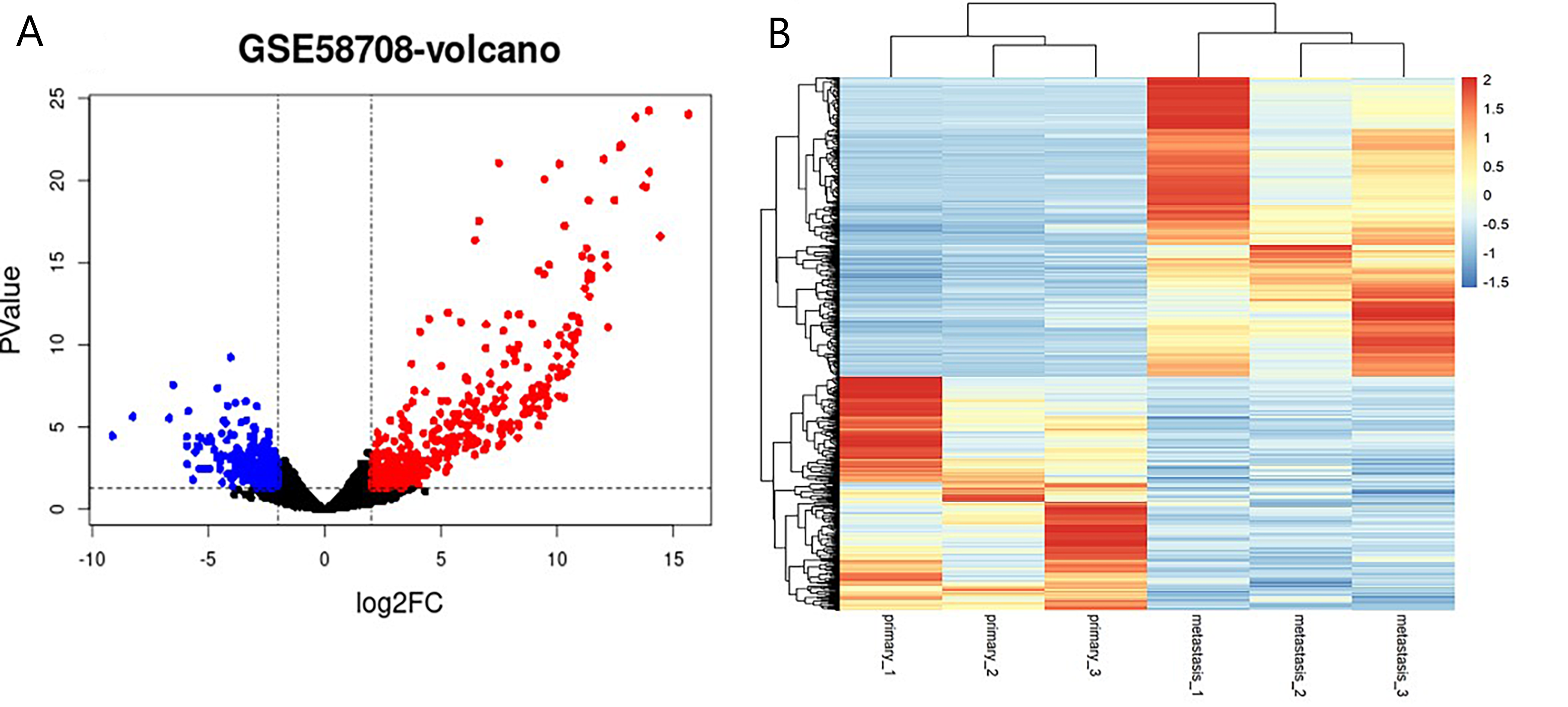

Supplement: Supplemental Information 3 — (A) volcano plot of the 683 DEGs, including 410 up-regulated and 273 down-regulated genes, blue indicates down-regulated DEGs, red indicates up-regulated DEGs, the cut-off criteria is adj.P.Val < 0.05 and —log.2FC— < 2.0. (B) Heatmap of the DEGs, blue indicates down-regulated DEGs, red indicates up-regulated DEGs. DEGs: differentially expressed genes [file peerj-11-15311-s003.png]

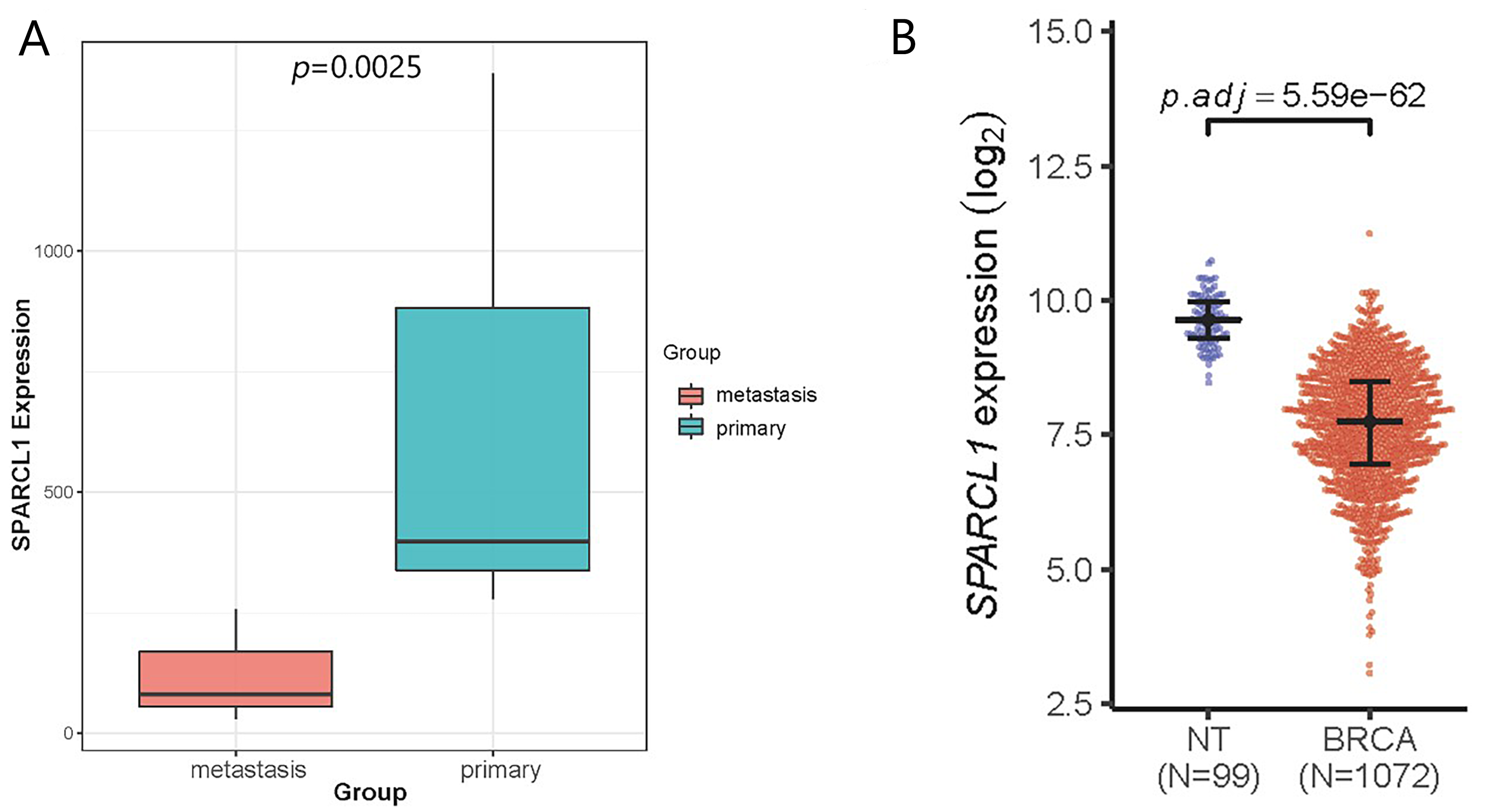

Supplement: Supplemental Information 4 — (A) The expression of SPARCL1 between liver metastasis and primary tissues in GSE58708. (B) The expression of SPARCL1 between BC tissues and normal tissues in TCGA. Metastasis: liver metastasis of BC, primary: primary tumor of BC, NT: normal tissue, BRCA: breast carcinoma, p.adj: adjusted p-value [file peerj-11-15311-s004.png]
